# Development of targeted adjuvants for HIV-1 vaccines

**DOI:** 10.1186/s12981-017-0165-8

**Published:** 2017-09-12

**Authors:** Jun Liu, Mario Ostrowski

**Affiliations:** 1 Clinical Sciences Division, University of Toronto, Room 6352, Medical Sciences Building, 1 King’s College Circle, Toronto, ON M5S1A8 Canada; 2 Department of Immunology, University of Toronto, Toronto, ON Canada; 3 Keenan Research Centre for Biomedical Science of St. Michael’s Hospital, Toronto, ON Canada

**Keywords:** HIV, Vaccine, Adjuvant, TNFSF, TLRs, NODs

## Abstract

Finding new adjuvants is an integrated component of the efforts in developing an effective HIV-1 vaccine. Compared with traditional adjuvants, a modern adjuvant in the context of HIV-1 prevention would elicit a durable and potent memory response from B cells, CD8^+^ T cells, and NK cells but avoid overstimulation of HIV-1 susceptible CD4^+^ T cells, especially at genital and rectal mucosa, the main portals for HIV-1 transmission. We briefly review recent advances in the studies of such potential targeted adjuvants, focusing on three classes of molecules that we study: TNFSF molecules, TLRs agonists, and NODs agonists.

## Background

More than three decades after human immunodeficiency virus 1 (HIV-1) was identified as the cause of AIDS, we still do not have an effective vaccine to stymie its global spread [1]. Barriers to developing an effective HIV-1 vaccine include the following: (1) HIV-1 mutates rapidly and has a tremendous genetic diversity. In this regard, broadly neutralizing antibodies (bNAbs) can neutralize a broad range of HIV-1 isolates, but we do not know how to induce such bNAbs with a vaccine [2]. Vaccines that induce non-broadly neutralizing HIV-1 Env-binding antibodies can afford partial protection against HIV-1/SHIV infection, but their efficacy needs to be substantially improved for clinical use [3, 4]. (2) All HIV-1 envelope (Env) based vaccine candidates can only induce a short-lived antibody response. This is in striking contrast to vaccines currently in clinical use and may severely limit the long-term efficacy of HIV-1 vaccines [5–8]. The mechanisms underlying this short duration of Env-antibody responses are not clear yet, but might be due to the failure of the Env glycoprotein to induce long-lived plasma cells [9, 10]. (3) HIV-1 is a rapidly replicating lentivirus that can establish latent infection soon after infection [11]. Thus an effective HIV-1 vaccine should elicit memory immune responses that can be mobilized fast (probably within a few days of infection) and sufficiently to block HIV-1 transmission through genital and rectal mucosa. Cytomegalovirus (CMV)-vectored HIV-1 vaccine might be able to elicit such a persistent and strong immune response [12], but we do not know if and how other vaccine platforms can elicit such immune responses, especially at genital and rectal mucosa. (4) CD4^+^ T cells play a pivotal role in forming memory immune response but are also target cells of HIV-1. An effective HIV-1 vaccine should induce potent cellular and humoral memory immune responses but avoid or limit stimulation of HIV-1 susceptible CD4^+^ T cells, which is highlighted by the Step and Phambili clinical trials results [13, 14]. Overcoming these barriers requires a multidisciplinary and multipronged approach, such as design of novel immunogens, development of better adjuvants, testing of multiple vaccination routes/schedules, and invention of novel delivery vehicles. Recent advances in immunology should be able to replace traditional adjuvants, such as alum, with an adjuvant that can preferentially promote protective responses from B cells, CD8^+^ T cells, and/or natural killer cells (NK), but not activate CD4^+^ T cells. Here, we will briefly review recent advances in the studies of such potential targeted adjuvants for HIV-1 vaccines. A thorough review is out of the scope of this short paper, and we will focus on three classes of molecules that we are studying: tumor necrosis factor superfamily (TNFSF) molecules, toll-like receptors (TLRs) agonists, and nucleotide-binding oligomerization domain-containing proteins (NODs) agonists.

## TNFSF molecules-CD40L, BAFF, and APRIL

TNFSF molecules are type II transmembrane proteins that have a conserved tumor necrosis factor homology domain at their C-termini [15]. Many TNFSF members are immune costimulatory molecules, among which CD40 ligand (CD40L), B cell activating factor (BAFF), and a proliferation-inducing ligand (APRIL) are pivotal for B cell costimulation. CD40L expressed on activated CD4^+^ T cells binds CD40 on B cells to promote B cell proliferation and survival, antibody isotype switching, and antibody affinity maturation. BAFF and APRIL are two closely related TNFSF molecules that are important for B cell development and differentiation [16, 17]. BAFF binds to three receptors on B cells: BAFF receptor (BAFFR), transmembrane activator and calcium modulator and cyclophilin ligand interactor (TACI), and B cell maturation antigen (BCMA) while APRIL binds to TACI and BCMA. BAFF–BAFFR interaction provides a key survival signal for mature B cells [16, 17]. The APRIL–BCMA pathway is essential for long-term survival of bone marrow plasma cells [18, 19]. BAFF and APRIL can also induce antibody isotype switching independent of CD40L [20]. Notably, BAFF and APRIL were shown to be essential for IgA production. The CD40L-CD40 pathway is also important for promoting the CD8^+^ T cell response. Binding of CD40 on immature DC by CD40L activates and matures them, which are “licensed” to activate CD8^+^ T cells.

Many reports have been published on testing CD40L as adjuvant for HIV-1 and Simian immunodeficiency virus (SIV) vaccines. We reported CD40L expressed from a canarypox vector (ALVAC) enhanced memory polyfunctional cytotoxic T cell (CTL) responses elicited by an ALVAC HIV-1 vaccine in mice [21]. Kwa et al. found CD40L augmented SIV-specific humoral and cellular immune responses, improved protection against SIV infection, and strengthened control of SIV replication in rhesus macaques receiving DNA prime/Modified Vaccinia Ankara (MVA) boost SIV vaccine [22, 23]. We recently found CD40L mainly enhanced SIV Env-specific antibody responses elicited by an ALVAC prime-Env protein boost SIV vaccine in monkeys (Liu et al. manuscript in preparation). Although further study is required, these results indicate CD40L could be a potential adjuvant capable of targeting B cells and CD8^+^ T cells.

BAFF and APRIL were also reported to enhance immunogenicity of HIV-1 vaccines. Gupta et al. found plasmid expressing multimeric soluble BAFF or APRIL, when co-administered with plasmid expressing IL-12, increased titer and avidity of gp120-binding antibodies and titer of neutralizing antibodies against a tier-1 and an autologous tier-2 HIV-1 virus in mice receiving a DNA prime/protein boost HIV-1 gp140 vaccine [24]. Melchers et al. made trimeric fusion constructs of HIV-1 gp140 with CD40L, BAFF, and APRIL and found only the gp140-APRIL construct significantly enhanced Env-binding antibodies in rabbits [25]. These previous reports just tested antibodies in blood. We found BAFF and APRIL increased HIV-1 Env-binding antibodies at mucosa in mice (Liu et al. manuscript in preparation).

## TLRs agonists

TLRs are type I transmembrane proteins belonging to pattern recognition receptors (PRRs), a large family of molecules that can sense “danger signals” (pathogen-associated molecular patterns and damage-associated molecular patterns) to activate innate immune cells, which then initiates adaptive immune responses through production of cytokines and chemokines and antigen presentation. Ten TLRs have been identified in human and 12 in mouse, each of which has distinct ligands [26]. Synthetic TLRs agonists, especially TLR7, TLR8, and TLR9 agonists, have been tested as adjuvants for HIV-1/SIV vaccines in animal studies. Moody et al. compared the effect of TLR4 agonist (lipid A), TLR7/8 agonist (R848), and TLR9 agonist (oCpG), either alone or in pairwise combination, on antibody responses elicited by a gp140 protein vaccine in monkeys [27]. They found combination of R848 and oCpG helped the vaccine induce the strongest Env-binding antibodies, including neutralizing antibodies and antibodies mediating antibody-dependent cell-mediated cytotoxicity (ADCC). Based on previous studies, the authors suggested combination of R848 and oCpG might enhance antibody responses by suppressing type 1 T helper cells (Th1). Kasturi et al. used combination of TLR4 and TLR7/8 agonist (MPL and R848) encapsulated in poly(lactic-*co*-glycolic acid) (PLGA) nanoparticles as adjuvant for SIV Env plus Gag protein vaccine or SIV virus-like particle (VLP) vaccine [28]. They reported that PLGA (MPL + R848) helped the SIV vaccine elicit persistently higher SIV Env binding IgG and IgA in blood and at mucosa, more long-lived Env-specific plasma cells in bone marrow and draining lymph nodes, and higher Env-specific CD4^+^ T cell responses than alum. Only PLGA (MPL + R848) adjuvanted SIV vaccines significantly protected monkeys expressing a restrictive tripartite motif-containing protein 5α (TRIM5α) allele from a heterologous SIV intravaginal challenge, and the protection correlated with SIV Env-binding IgG in blood and vaginal secretion.

We recently reported that self-assembling peptide nanofibers could co-deliver an HIV-1 CD8^+^ T cell epitope, SL9, and TLR7/8 agonist R848 to activate human monocyte-derived dendritic cells (MDDCs) in vitro and elicited stronger SL9-specific CD8^+^ T cells in HLA-A2 transgenic mice [29]. EAK16-II is a 16mer peptide that can self-assemble to form nanofibers in aqueous solution. We found SL9-EAK16-II fusion peptide could co-assemble with R848 and TLR7 agonist R837 to form nanofibers. The nanofibers were taken up by MDDCs into endosomes, where TLR7 and TLR8 are localized. Consequently, SL9-EAK16-II nanofibers with R848 or R837 activated MDDCs, which elicited stronger SL9-specific CD8^+^ T cell responses in vitro than non-nanoformed SL9 peptide. R848 was more potent than R837 in helping the nanofibers to induce the SL9-specific CD8^+^ T cell responses in vitro, possibly due to its synergistic activation of both TLR7 and TLR8 in DCs. The mechanisms underlying the enhanced SL9-specific CD8^+^ T cell induction by SL9-EAK16-II nanofiber in vitro and in vivo are still under investigation, but are possibly related to its increased stability due to resistance to extracellular and intracellular proteinases and peptidases (Liu et al. unpublished data).

## NODs agonists

NODs are intracellular PRRs [30]. There are two closely related NODs, NOD1 and NOD2, all of which containing N-terminal caspase recruitment domain(s) (CARD) (one for NOD1 and two for NOD2) to activate downstream signaling molecules, a C-terminal leucine-rich repeat domain to recognize microbial molecules, and a central nucleotide-binding oligomerization domain to bind nucleoside triphosphate. The ligands of NODs are components of peptidoglycan in bacterial cell wall. NOD1 ligand is γ-d-glutamyl-mesodiaminopimelic acid (iE-DAP) present in some Gram-positive bacteria and all Gram-negative bacteria. NOD2 ligand is muramyl dipeptide (MDP) found in all Gram-positive and Gram-negative bacteria. These ligands bind and activate NODs, which finally activates nuclear factor kappa-light-chain-enhancer of activated B cells (NF-κB) and activator protein 1 (AP-1), leading to autophagy and production of pro-inflammatory cytokines, chemokines, and anti-microbial factors. Activation of NOD1 and NOD2 primes a Th2-polarized adaptive immune response with potent antibody responses in mice [31], which makes NODs agonists attractive as adjuvants for HIV-1 vaccines, since Th2 cells are much less susceptible to HIV-1 infection than Th1 and Th17 [32]. Pavot et al. reported NOD1 and NOD2 agonists encapsulated in polylactic acid (PLA) nanoparticles enhanced mucosal antibody responses elicited by HIV-1 p24 coated on PLA nanoparticles in mice [33]. Both NOD1 and NOD2 agonists augmented p24-specific IgG in feces after subcutaneous vaccination, compared with p24-alum or PLA-p24. Only NOD2 agonist significantly enhanced p24-specific IgA in feces and vaginal lavage after oral or intranasal vaccination, respectively, and p24-specific IgG in vaginal lavage after intranasal vaccination. These findings suggest NOD2 agonist may be better than NOD1 agonist as an adjuvant to elicit mucosal antibody responses. We found MDP could enhance mucosal gp140-specific antibody response in mice (Liu et al. unpublished data).

## Conclusions and perspectives

Recent advances in the development of targeted adjuvants should help HIV-1 vaccines elicit potent and durable memory responses of B cells, CD8^+^ T cells, NK cells, etc. while avoiding generation of abundant HIV-1 susceptible CD4^+^ T cells at genital and rectal mucosa. An ideal adjuvant should preferentially activate B cells, CD8^+^ T cells, and NK cells other than CD4^+^ T cells. Using targeted delivery vehicles, such as nanoparticles coated with specific ligands for the receptors on these cells, may further increase the targeting of the adjuvants. More studies are still needed to find the best targeted adjuvant for HIV-1 vaccine before clinical trials.

## Abbreviations

ADCC: antibody-dependent cell-mediated cytotoxicity
AP-1: activator protein 1
APRIL: a proliferation-inducing ligand
BAFF: B cell activating factor
BAFFR: BAFF receptor
BCMA: B cell maturation antigen
bNAbs: broadly neutralizing antibodies
CARD: caspase recruitment domain
CD40L: CD40 ligand
CMV: cytomegalovirus
CTL: cytotoxic T cell
CXCL10: C-X-C motif chemokine 10
DCs: dendritic cells
Env: HIV-1 envelope protein
HIV-1: human immunodeficiency virus 1
iE-DAP: γ-d-glutamyl-mesodiaminopimelic acid
MDDC: monocyte-derived dendritic cells
MDP: muramyl dipeptide
MVA: Modified Vaccinia Ankara
NF-κB: nuclear factor kappa-light-chain-enhancer of activated B cells
NK: natural killer cells
NODs: nucleotide-binding oligomerization domain-containing proteins
PLA: polylactic acid
PLGA: poly(lactic-*co*-glycolic acid)
PRRs: pattern recognition receptors
SHIV: Simian-human immunodeficiency virus
SIV: Simian immunodeficiency virus
TACI: transmembrane activator and calcium modulator and cyclophilin ligand interactor
TLRs: toll-like receptors
TNFSF: tumor necrosis factor superfamily
TRIM5α: tripartite motif-containing protein 5α
VLP: virus-like particle
